# Visual Analytics for Epidemiologists: Understanding the Interactions Between Age, Time, and Disease with Multi-Panel Graphs

**DOI:** 10.1371/journal.pone.0014683

**Published:** 2011-02-15

**Authors:** Kenneth K. H. Chui, Julia B. Wenger, Steven A. Cohen, Elena N. Naumova

**Affiliations:** 1 Department of Public Health and Community Medicine, Tufts University School of Medicine, Boston, Massachusetts, United States of America; 2 Division of Nephrology, Massachusetts General Hospital, Boston, Massachusetts, United States of America; 3 Department of Civil and Environmental Engineering, Tufts University School of Engineering, Medford, Massachusetts, United States of America; University of Michigan, United States of America

## Abstract

**Background:**

Visual analytics, a technique aiding data analysis and decision making, is a novel tool that allows for a better understanding of the context of complex systems. Public health professionals can greatly benefit from this technique since context is integral in disease monitoring and biosurveillance. We propose a graphical tool that can reveal the distribution of an outcome by time and age simultaneously.

**Methodology/Principal Findings:**

We introduce and demonstrate multi-panel (MP) graphs applied in four different settings: U.S. national influenza-associated and salmonellosis-associated hospitalizations among the older adult population (≥65 years old), 1991–2004; confirmed salmonellosis cases reported to the Massachusetts Department of Public Health for the general population, 2004–2005; and asthma-associated hospital visits for children aged 0–18 at Milwaukee Children's Hospital of Wisconsin, 1997–2006. We illustrate trends and anomalies that otherwise would be obscured by traditional visualization techniques such as case pyramids and time-series plots.

**Conclusion/Significance:**

MP graphs can weave together two vital dynamics—temporality and demographics—that play important roles in the distribution and spread of diseases, making these graphs a powerful tool for public health and disease biosurveillance efforts.

## Introduction

Visual analytics enhances the understanding of large, complex data with effective, interactive visualization [1]. The technique can be especially useful in epidemiological investigations, which involves monitoring trends, detecting anomalies, and generating testable hypotheses. The potential of visual analytics in epidemiology has been recently illustrated by a number of applications, including flow-maps to display population mobility and person-to-person contact across wide geographic space [2], dynamic mapping of disease occurrences with simultaneous depiction of spatio-temporal changes in environmental factors [3], detection of spatiotemporal hotspots [4], and displaying genotype data [5]. Ongoing federally-funded programs for implementation of these visual analytic techniques in support of pandemic planning and response may further stimulate interest in visual analytics [6]. Although the application of visual analytics in biosurveillance has not been extensively studied, new uses for these techniques could lead to important breakthroughs in surveillance applications. The growth of surveillance systems in both quantity of data and variety of outcomes is likely to necessitate constant innovations in data processing, synthesis, and communication [7].

The increasing popularity of graphical applications for data visualization has inspired a growing body of diverse graphs, charts, plots, and maps in research literature. Impressive attempts have been made to guide proper construction and interpretation of visual displays [8], [9], [10], [11]. However, specific recommendations for using complex graphs in epidemiological studies are lacking.

In this paper we present examples of how visual analytics can be used by epidemiologists and public health professionals to facilitate data interpretation and decision-making. We provide instructions for building plots consisting of multiple panels and offer guidance for properly interpreting information abstracted from these graphs. We also provide justification for the use of rates, detailed age pyramids, and refined temporal scales in visual analytics.

## Methods

### Data Sources

The data sets used in this analysis represent different time periods, geographic levels, demographic characteristics, and disease measurements. To illustrate the use of MP graphs for nationally representative data we utilized the Medicare database. The database, maintained by the Centers for Medicare and Medicare Services (CMS), covers over 96% of US residents aged 65 years and older, representing about 36 million residents [12]. We abstracted all hospitalizations that involved influenza (ICD-9-CM code 487, n = 250,858) and salmonellosis (ICD-9-CM code 003, n = 27,950) between January 1991 and December 2004. We compiled a weekly time-series data set (728 weeks) with counts of influenza and salmonellosis and estimated disease rates per one million population aged 65 or above. Population denominators for these rates were calculated using population estimates derived from the United States Census Bureau Population Estimates Program for individual years, then linearly interpolated for each week of the study period.

To illustrate the use of MP graphs on a regional level we used state surveillance records from Massachusetts (population 6.4 million). Confirmed cases of salmonellosis (n = 2,287) for the years 2004–2005 were abstracted from case reports submitted to the Massachusetts Hinton State Laboratory Institute of the Massachusetts Department of Public Health (MDPH). Case definitions and data collection methods for this dataset have been described in detail elsewhere [13], [14]. To illustrate the use of MP graphs for community level data, we abstracted 15,508 records of hospital visits for asthma among children aged 0–18 years from the billing data of emergency department, outpatient visits, and inpatient admissions to Milwaukee Children's Hospital of Wisconsin (CHW) for the years 1997–2006. CHW is the primary pediatric referral hospital for children aged 0–18 years in eastern Wisconsin and represents 85% of all hospital admissions in Milwaukee County for children aged 0–19 years [15]. All analytical results and graphs were produced using SPSS version 17.0 and S-Plus 8 for Windows.

### Ethics Statement

This is a secondary data analysis. The protocol was approved by the Tufts Institutional Review Board. Approvals for the use of these data sets were obtained from the relevant Institutional Review Boards and data owners. All data were de-identified before being delivered to the authors for analysis.

### Visualization and Analysis

In this section we describe the important features of population pyramids, time-series plots, and image plots and then go on to explain the compilation of an MP graph.

### Outcome Pyramids

The outcome pyramid is based on the population pyramid, which is a type of graph commonly used to describe the composition of age and sex of a population. Population pyramids contain two vertically juxtaposed histograms, one for males and the other for females with a common vertical axis for age, which is usually represented by single years or 5-year categories. The longer a bar extends from the vertical axis, the greater the proportion or number of individuals in that age category. The horizontal axis can also be modified to depict disease counts and rates, what we refer to as an “outcome pyramid” in this paper.

In assessing outcome pyramids, attention should be paid to: 1) the general shape of a pyramid, such as uniform or triangular; 2) the presence or absence of symmetry by gender; and 3) irregularities and specific features, such as bumps or spikes in cases. Outcome pyramids themselves provide an important visual tool for assessing age and gender differences in diseases. However, static outcome pyramids are insensitive to temporal changes. These changes can be substantial, especially for infectious diseases with well-pronounced seasonality [16], [17], [18].

### Time-series Plots

Time-series plots visualize temporal trends and seasonal patterns in disease rates. An informative time-series plot must contain a sufficiently long time frame and a carefully chosen unit of time, such as day, week, month, or year. While various levels of temporal aggregation have both benefits and limitations, a weekly time-series is standard for many surveillance systems [19]. Like the horizontal axis for outcome pyramids, the vertical axis of a time-series plot can display either disease counts or rates.

While a well-constructed time-series plot reflects temporal fluctuations in disease occurrence, interpretation of such fluctuations rests on the assumption of equal risks across the represented age categories. However, diseases rarely affect all ages uniformly: older adults and infants often experience higher age-specific disease rates. This can be partly alleviated by compiling time-series plots for specific age groups. However, small age group intervals (e.g. single year) lead to an overwhelming number of plots, while medium sized intervals (e.g. 5-year) still require the aforementioned assumption of equally distributed risks within the five years interval. Therefore, better integration of demographic information into time-series plots is needed to more accurately interpret disease trends. Although outcome pyramids and time-series plots alone are useful for surveillance purposes, they are still two separate constructs and hence cannot be used to observe temporal-demographic interaction on their own [20].

### Image Plots

An image plot is capable of displaying information for at least three variables. The first two variables are shared and represented by the horizontal axis and vertical axis, which form a grid of numerous rectangular tiles. In our example, these two variables are time and age. The third variable is represented by different hues or saturations of colors in the rectangular tiles. In our examples, the third variables are disease rates or counts.

**Figure 1:**
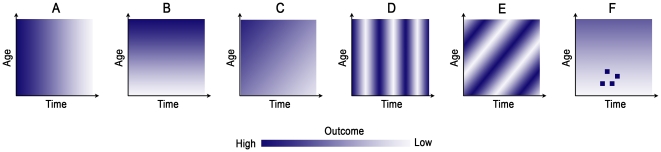
Typical patterns observed in image plots used to study the association between age, time, and the disease of interest.

New information previously masked by case pyramids and time-series plots can be revealed from image plots. However, reading the graph properly requires some training and practice. Figure 1 displays some of the typical patterns. In this figure, a monochromatic color scale is used, with higher saturation, or darker colors, representing higher values of an outcome. Panel A shows a decrease of color saturation from left to right, indicating that the outcome decreases along time, and the observed decrease is somewhat uniform across age. Panel B shows a decrease of color saturation from top to bottom, indicating an increase in an outcome in the older age group irrespective of time. Panel C shows the combined effect of Panels A and B: an outcome increasing with age but decreasing across time. Panel D shows a striated pattern typical for periodic fluctuations in an outcome across time—evidence of seasonality. Panel E also shows a striated pattern, but tilted at an angle. This pattern indicates that at any time point, the outcome across age is uneven and likely to be cohort-specific. This phenomenon, known as the “age-cohort effect,” [21] can be observed if an outcome was measured in the same cohort repeatedly and an outcome remains higher or lower in a group of subjects as they aged. Finally, Panel F shows a set of four distinct clusters combined with Panel B, where substantially higher outcomes in the younger group at the middle of the time duration are observed. These clusters might be indicative of disproportionally high values of an outcome, or an aberration in the expected values. Depending on the context a cluster, the pattern may indicate potential outbreaks.

### The Construction of a Multi-panel Graph

We propose a new graphical design called a multi-panel (MP) graph, which involves the strategic positioning of two or more graphs sharing at least one common axis on a single canvas. In our example, we propose combining the time-series plot, outcome pyramid, and image plot by aligning them together so that they share a common set of axes that represent age and time. This design allows for the simultaneous visualization of population structure and temporal trend, which is important for us to clearly understand trends in disease burden and, more importantly, for infectious diseases, to quickly detect historical outbreaks [22], [23].

## Results

**Figure 2:**
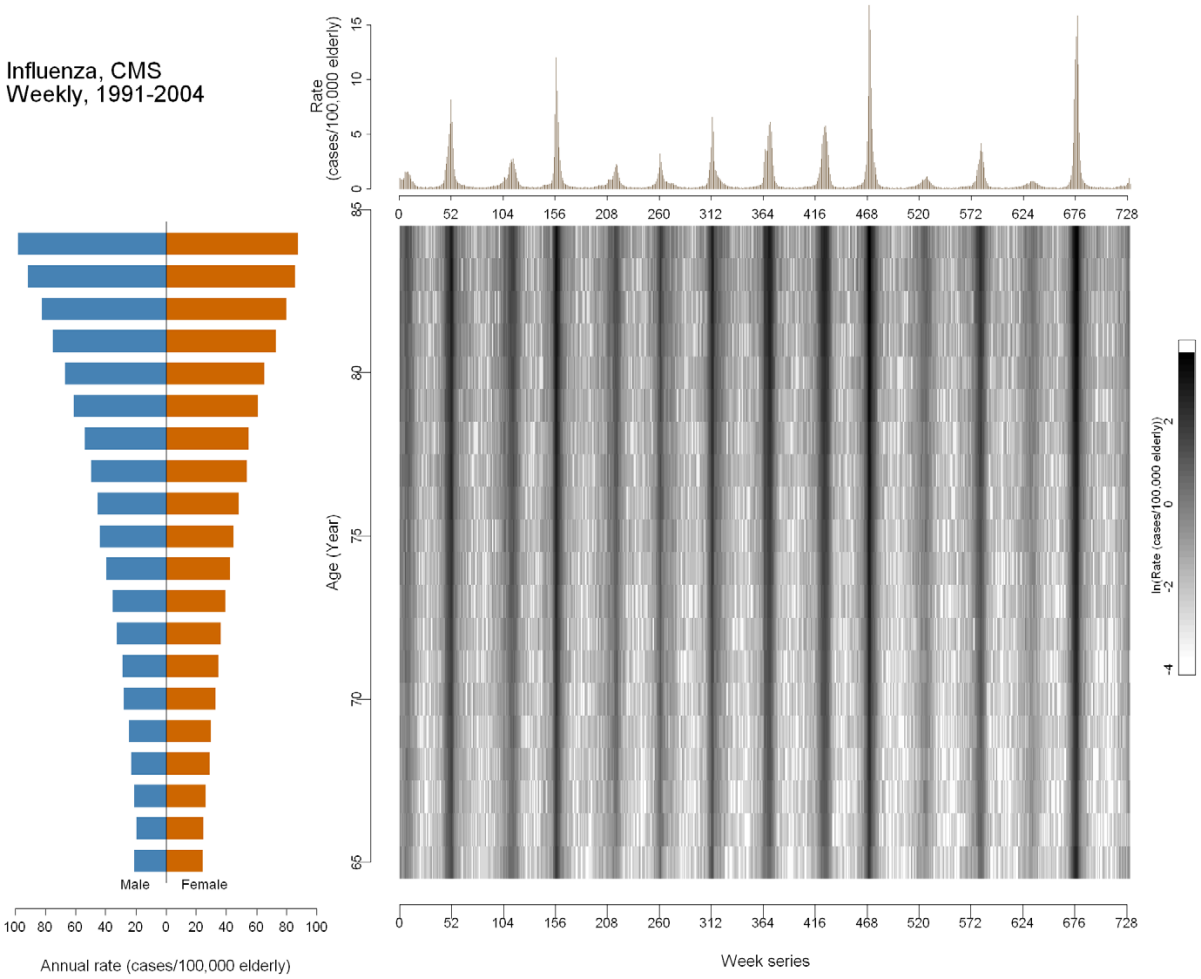
Multi-panel graph of influenza in the United States older adult population (aged 65 and over) 1991–2004. Lower left: outcome pyramid; upper right: time-series plot; lower right: image plot.

We first demonstrate the use of the MP graph with a disease that has a well pronounced seasonal pattern. Figure 2 shows influenza-associated hospitalizations among 65–85 year olds in the United States, 1991–2004. The outcome pyramid depicts the rates for each single year of age by gender. The horizontal bars lengthen with increasing age, indicating that the rates of influenza increase with age. The upper time-series shows rates plotted against time, expressed in weekly units. The ticks of the horizontal axis are set at every 52^nd^ week as an approximation of one calendar year. The vertical, regularly occurring spikes near the beginning of each year represent the peaks of influenza epidemics. In some years, the spikes are particularly high (1999–2000, week 468; 2003–2004, week 676), indicating more severe epidemics. The image plot displays the log-transformed rate projected onto an age-by-time grid, allowing the examination of the age-time interaction. The data were log-transformed to improve visualization of the whole range of values due to right-skewness in the distribution of rates. Two important features are shown in the image plot. First, vertical strips corresponding to the seasonal peaks shown in the time-series indicate an annual seasonal pattern. Second, for most of the dark strips, the color is more saturated for the older age groups, illustrating a higher rate of disease. In severe epidemic years, the saturation of the strips is more uniform, indicating substantial rate increases in all age groups. The lighter regions between the dark strips represent the “off-season”, commonly defined as April through September. During the off-seasons, the rates are also higher for the older age groups compared to the younger groups. Among those lighter areas, the lower right corner of the strips appears to be lighter than the upper left corner, suggesting a possible cohort effect.

**Figure 3:**
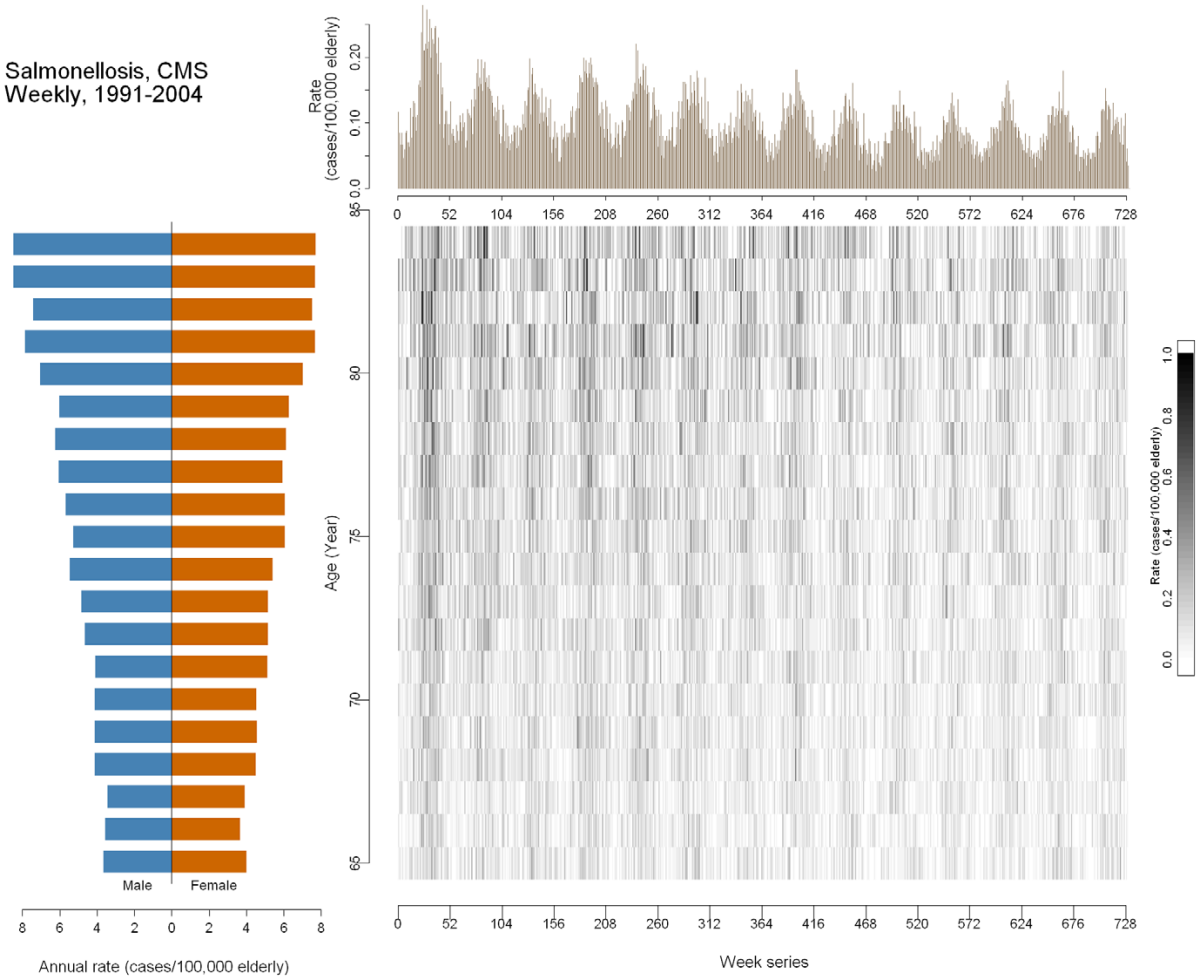
Multi-panel graph of salmonellosis in the United States older adult population (aged 65 and over) 1991–2004. Lower left: outcome pyramid; upper right: time-series plot; lower right: image plot.

Figure 3 shows information on hospitalizations associated with non-typhoidal salmonellosis in the older adult US population ages 65–85 years, 1991–2004. The outcome pyramid shows a higher rate of disease for the older population. The time-series plot shows an oscillatory pattern which is less extreme than that of influenza. The peaks occur during the middle of each year, typically during the summer months in the US, which agrees with findings of other investigations [18], [24], [25]. There is also a declining long-term trend from 1991 through 1998, and then the trend stabilizes in 1999 (week 416). The image plot shows the rate by age and week. The vertical strips associated with the seasonal pattern are less profound compared to that of influenza, but are still discernable. The younger group, once again, has a lower rate. The rates are also higher at the top left corner compared to the lower right. A cohort effect may be possible, but other measures such as new food safety regulation or improved food safety knowledge could also have contributed to the observed decline [26].

**Figure 4:**
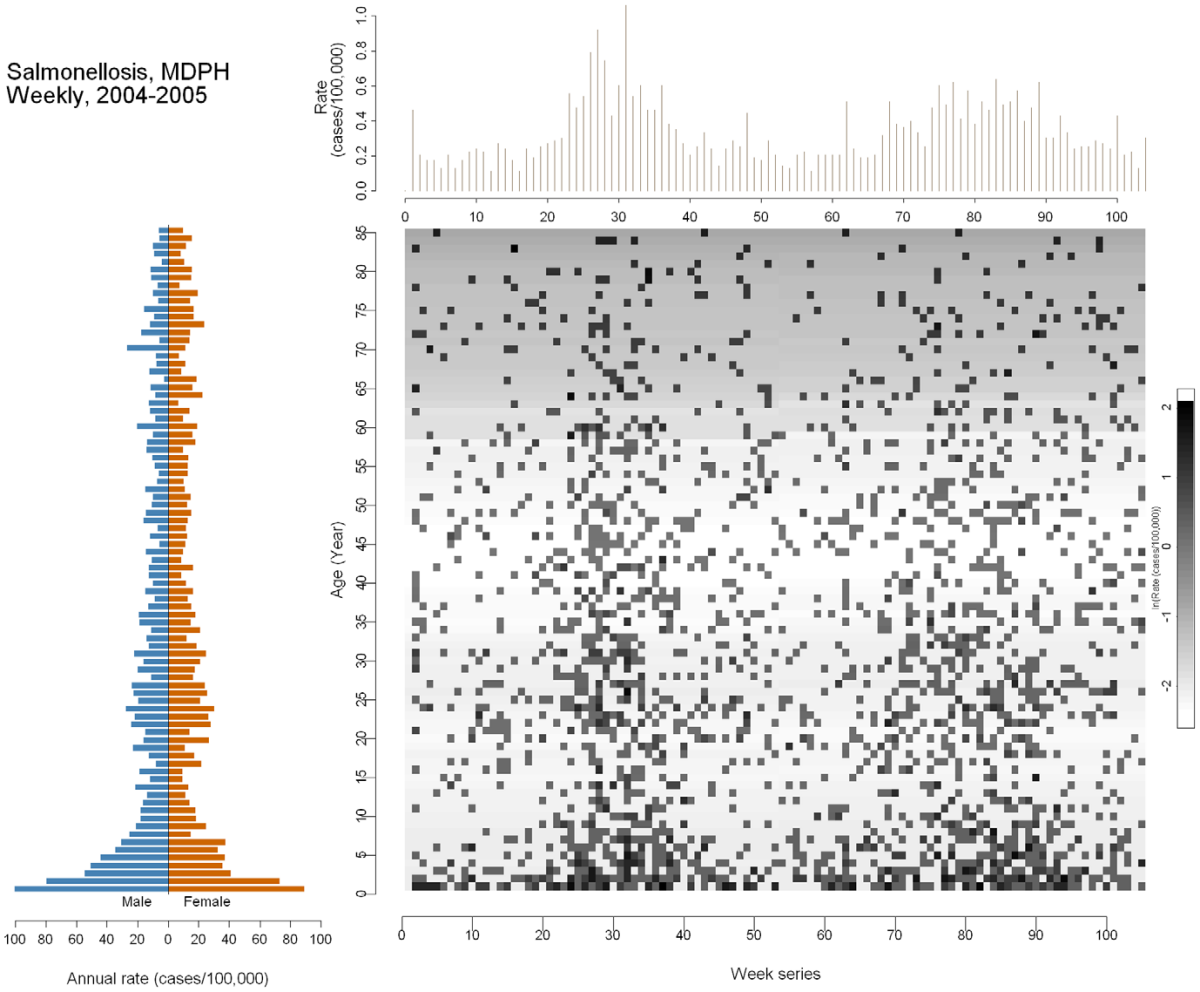
Multi-panel graph of salmonellosis in the general Massachusetts population 2004–2005. Lower left: outcome pyramid; upper right: time-series plot; lower right: image plot.

Figure 4 shows information on reported cases of salmonellosis in Massachusetts, 2004–2005. The outcome pyramid displays number of cases per 100,000 persons by gender. Infants and children ≤8 years old seem to be most at risk, as represented by the long horizontal bars. Another small peak occurs during early adulthood (18–30 years old). This observed increase during early adulthood may be attributable to insufficient knowledge of food hygiene, indirectly increasing the risk of food poisoning during eating out, cooking, or inter-household infection involving young children [27]. The time-series plot shows two seasonal peaks: between the 25^th^ and 35^th^ weeks (summer, 2004) and a smaller one between the 75^th^ and 90^th^ weeks (summer, 2005). The image plot is more pixelated compared to the previous ones because the range of time has dropped from 728 weeks to 104. Even so, the vertically aligned clusters can still be clearly distinguished. The light shade of grey in the background for those aged 65 years and above indicates a low rate of *Salmonella* infection across the two years, although infants, children, and young adults contribute the majority of the cases.

**Figure 5:**
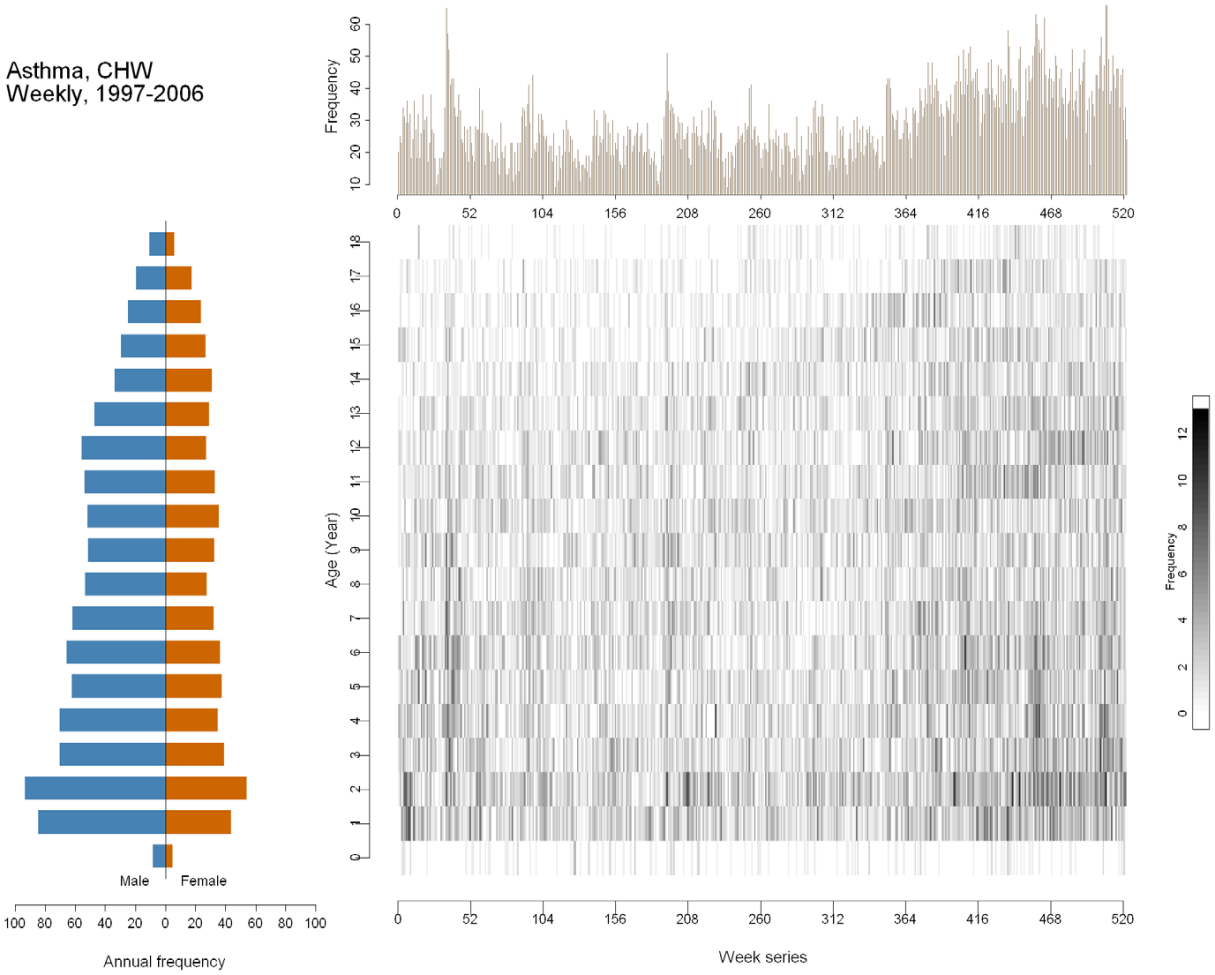
Multi-panel graph of asthma in children aged 0–18 in Milwaukee 1997–2006. Lower left: outcome pyramid; upper right: time-series plot; lower right: image plot.

Finally, Figure 5 shows cases of childhood asthma in Milwaukee, Wisconsin during the period 1997–2006. This figure differs from the other three in that it shows counts rather than rates due to the lack of a proper regional denominator in the Milwaukee area. The outcome pyramid shows several important characteristics. First, there were fewer cases at age 0. The numbers of asthma cases are highest for toddlers aged 1–2 years old. There were more male cases than female cases, with a male-to-female ratio ranging from 1.5 to 2, although the ratio decreases as age increases. The time-series plot shows a non-uniform seasonal pattern: the winter months had slightly higher asthma cases than the summer months did. The overall number of visits due to asthma increased markedly, particularly over the last three to four years of observation. Many potential factors could have caused the observed increase. First, the time-series only shows frequency, so the increase might be due to changes in the size of the selected population. For instance, increased immigration to Milwaukee, closing of a nearby hospital, or expansion of the hospital, both in size and service, could have caused the observed increase. The image plot reveals that the increase in asthma cases did not occur uniformly throughout all of the age groups. Except for the spike in late 1997, most of the cases occurring in children aged 4–12 seem to stabilize from 1997 to 2004. After 2004, marked increases in frequency are seen in the 2 to 6 year old age group, forming a darker shade at the lower right corner.

## Discussion

Technological advancements have enabled health-related data collection and storage in larger scale and higher frequency. This has led to the compilation of a tremendous amount of information, which is challenging for analysts to sort through. With the growth of epidemiologic research examining climate change, population growth, migration, emerging pathogens and natural disasters, monitoring human health through surveillance data is becoming increasingly complex. Epidemiologists can benefit immensely from adopting innovative visual-analytical techniques used in other fields of study. This includes the utilization of population pyramids by demographers to describe the age structure of a community, along with modified Lexis diagram surfaces to describe incidence patterns in diseases, such as influenza, simultaneously by age, period, and cohort. Time-series plots have also been widely used in economics and physics. By strategically combining these visualization techniques in an MP graph, we can more efficiently analyze surveillance data through discerning interactions between variables, detecting anomalies in the data, identifying temporal trends, and discovering other nuances. In this section we discuss some potential analytical recommendations stemmed from the findings in the MP graphs, as well as the strengths of MP graphs and caveats when applying this technique.

### Seasonality of influenza in the U.S

A well-constructed MP graph can inform the decisions used in statistical modeling. The time-series plot in Figure 2 shows that influenza hospitalizations exhibited a pronounced annual seasonal pattern: every winter there is a noticeable spike in influenza. To properly model this oscillation, a harmonic regression model can be employed:
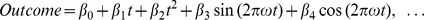
(i)where *t* represents time and *ω* represents the frequency (1 divided by the total number of time units in a cycle). The coefficients *β*_3_ and *β*_4_ in the equation (i) can be used to derive seasonal parameters such as time to peak and magnitude. Using this model, we found that the average peak week of influenza was the 28.6^th^ week (95% CI: 28.4, 28.8) or the 3^rd^ week of January. The median absolute intensity (outcome at peak – outcome at nadir) was 48.3 cases per one million older adults (95% CI: 45.8, 51.0) [28]. From Figure 2, we can also estimate the time to peak by looking for the highest point in the time-series plot or the darker strip in the image plot. The annual alternating contrast between the light and dark saturations allows us to estimate the absolute intensity as well. Notably, more intense flu seasons tend to peak earlier in the year, as shown in the uneven spacing of peaks with different heights and strips with different color saturations in the time-series plot and image plot, respectively. In fact, absolute intensity and peak timing had a strong, inverse relationship (Spearman's correlation coefficient = −0.5, p<0.05).

### Effectiveness of a food safety regulation enforcing testing for Salmonella

Other than providing improvements for statistical models, an MP graph can also help generate new hypotheses. For instance, we observed a decreasing trend in salmonellosis-associated hospitalizations, like the one shown in Figure 3, around the 300^th^ week (approximately early 1997), which then led to investigations into reasons for such a prominent drop. A potential reason could be the implementation of a nation-wide quality control program known as Hazard Analysis Critical Control Points (HACCP) in the broiler chicken production facilities. This hypothesis can be tested using the general model:
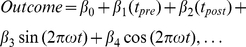
(ii)where again *t* represents time and *ω* represents frequency. The linear terms, *t_pre_* and *t_post_*, represent the time (in weeks) before and after the HACCP implementation began in early 1997. By comparing the regression coefficients of the two terms, we can estimate the possible effect of the HACCP regulation. We found that when looking at the whole nation, the pre-HACCP and post-HACCP rates of change did not differ. The rates of change in hospitalization associated with *Salmonella* before and after HACCP are −0.0009 (95% CI: −0.0018, −0.0001) cases per million people aged 65+ per week and −0.0009 (95% CI: −0.0015, −0.0002), respectively (p-value for the difference between slopes = 0.92). A decreasing trend was observed in salmonellosis-associated hospitalizations, but the HACCP regulation did not seem to be associated with a steeper decrease in salmonellosis-associated hospitalizations after it was implemented. A closer investigation by Census division (results not shown) revealed that the effect of HACCP in preventing *Salmonella* infections could have been modified by geographic area: The southern part of the US did not demonstrate any beneficial post-HACCP change [26].

### Strengths of MP Graphs

Susceptibility to infectious diseases varies by age, time, and geography. Understanding the interactions of these three elements is important for prediction and prevention of disease outbreaks in vulnerable populations. We have adopted and advanced a set of traditional graphical tools for visualizing demographic and temporal patterns simultaneously. As illustrated by the four examples, the MP graph presents a straightforward and effective way of visualizing key temporal and age-related trends that might otherwise be hidden if the traditional visualization techniques were used individually.

The component graphs themselves require only standard statistical packages and basic knowledge of statistical analysis. By adding detailed statistical assessments, these graphs can be used for observational and analytical surveillance purposes. For instance, we can detect age-specific clusters and cohort effects when individuals of the same age at the same time exhibit differential disease patterns over the period of surveillance than other individuals of different ages [29]. As an extension, the tools can facilitate the detection of increased risk of disease in vulnerable subpopulations at a given time period, and depict the projected change in demographic structures and expected disease outcomes based on local forecasts. Although we have demonstrated the use of multi-panel graphs in retrospective surveillance data, the techniques can be further expanded by plotting projected trends, marking the time and age groups where deviations from expected values are noticed. The MP graph can be also animated and projected in real-time.

### Precautions in Using MP Graphs

There are some precautions when composing MP graphs. To build MP graphs properly, we have to consider the 1) precision of age valuation in describing population structure, 2) temporal scale and resolution, and 3) amplitudes of temporal variations in both populations at risk and the disease. Traditionally, the primary approach used to evaluate age-specific rates is to stratify the population into age categories and then to observe how the disease of interest affects each age group. A common example is the division of the population into three broad age categories: children, adults, and older adults [30], [31], [32]. Such an approach can cause problems if the age range for each category is set arbitrarily, as critical information may be lost during aggregation. The disadvantage of this approach is magnified if the disease of interest has different prevalence across age ranges that are finer than the chosen age categories [33]. Some diseases are more prevalent in children, while others are more prevalent in the older population. For example, the incidence of salmonellosis in 2003 in the United States is greatest among infants aged <1 year (122.7 per 100,000 infants) and second highest among children aged 1–4 years (50.6 per 100,000 children) [34]. Depending on the disease of interest, different age groups can be affected by the disease differently. In biosurveillance systems, the cases are typically shown without correction for the size of the affected population or the population at risk [23]. Interpretation of the “hot spots” in such situation should be done with caution, since the age-time interactions can be easily distorted due to skewing distributions toward age-specific population or unequal sizes of age groups. Our previous work has revealed significant changes in population profiles during various phases of seasonal oscillations in enteric infections [35].

Second, the seasonality of certain diseases can also be a concern in rate calculations, specifically for infectious diseases. In temperate climates, seasonal peaks of enteric disease are most likely to be observed in the summer months [36], [37], [38], [39], [40] while influenza and other respiratory infections are most prominent with the advent of the winter months [20], [41]. The shape and magnitude of temporal variations can be better understood if records cover a sufficiently long time period and represent a clearly-defined geographic area. Systematic over- and under-estimations of daily, weekly, monthly or yearly rates can occur if the population was erroneously assumed to be static. Potential sources of error in existing surveillance systems include variations in system specifications, data collection protocol, time periods over which the data are collected, and analytic methods applied to determine disease trend or departure from a typical norm.

### Future developments

MP graphs are a potentially useful tool for illustrating data with complex, multifaceted structure. Combined with live data streams, we can create an efficient dashboard device for the purpose of real-time surveillance. Two other features, if incorporated, can further enhance the power of the MP graph: dynamic display and interactivity. Dynamic display of data, such as a movie with consecutive snapshots showing the geographic distribution of an epidemic [3], allows for a better perception of disease progression. Such animation devices can also be applied to MP graphs. Interactive features such as user-controlled functions in enlarging or diminishing the scale, setting the lower and upper limits of the axes, or freely assembling different graphical components can make the MP device more versatile, improving its applicability.

### Conclusion

Increasing complexity in epidemiological data calls for more sophisticated graphical representations. Other than developing or adapting new visualization schemes, we can also enrich the number of variables, or dimensions, on a canvas by combining simple graphical modules. MP graphs offer a novel way to synthesize complex visual data. Further research into expanding the use of these techniques will help epidemiologists better identify the most vulnerable populations and times, enhancing the detection and prevention of diseases.

## References

[1] Thomas JJ, Cook KA (2005). Illuminating the Path: The Research and Development Agenda for Visual Analytics: National Visualization and Analytics Ctr.

[2] Guo D (2007). Visual analytics of spatial interaction patterns for pandemic decision support.. International Journal of Geographical Information Science.

[3] Castronovo DA, Chui KKH, Naumova EN (2009). Dynamic maps: a visual-analytic methodology for exploring spatio-temporal disease patterns.. Environmental Health.

[4] Maciejewski R, Rudolph S, Hafen R, Abusalah AM, Yakout M (2010). A Visual Analytics Approach to Understanding Spatiotemporal Hotspots.. Ieee Transactions on Visualization and Computer Graphics.

[5] Kumasaka N, Nakamura Y, Kamatani N (2010). The textile plot: a new linkage disequilibrium display of multiple-single nucleotide polymorphism genotype data.. PLoS One.

[6] Bisset K, Marathe M (2009). A Cyber Environment to Support Pandemic Planning and Response.

[7] Thacker SB, Berkelman RL (1988). Public health surveillance in the United States.. Epidemiol Rev.

[8] Cleveland WS (1993). Visualizing Data..

[9] Cleveland WS (1994). The Elements of Graphing Data..

[10] Tufte ER (2001). The Visual Display of Quantitative Information, 2nd edition..

[11] Wilkinson L (1999). The Grammar of Graphics..

[12] Cohen SA, Naumova E (2009). Population Dynamics in the Elderly: The Need for Age-Adjustment in National BioSurveillance Systems..

[13] Naumova EN, Chen JT, Griffiths JK, Matyas BT, Estes-Smargiassi SA (2000). Use of passive surveillance data to study temporal and spatial variation in the incidence of giardiasis and cryptosporidiosis.. Public Health Reports.

[14] Naumova EN, MacNeill IB, Auget J-L, Balakrishnan N, Mesbah M, Molenberghs G (2006). Sesonality assessment for biosurveillance systems.. Advances in Statistical Methods for the Health Sciences. 1st ed.

[15] Henrickson KJ, Hoover S, Kehl KS, Hua W (2004). National disease burden of respiratory viruses detected in children by polymerase chain reaction.. Pediatr Infect Dis J.

[16] Altizer S, Dobson A, Hosseini P, Hudson P, Pascual M (2006). Seasonality and the dynamics of infectious diseases.. Ecol Lett.

[17] Fisman DN (2007). Seasonality of infectious diseases.. Annu Rev Public Health.

[18] Naumova EN, Jagai JS, Matyas B, DeMaria A, MacNeill IB (2007). Seasonality in six enterically transmitted diseases and ambient temperature.. Epidemiology and Infection.

[19] Choi K, Thacker SB (1981). An evaluation of influenza mortality surveillance, 1962-1979. I. Time series forecasts of expected pneumonia and influenza deaths.. Am J Epidemiol.

[20] Lofgren E, Fefferman NH, Naumov YN, Gorski J, Naumova EN (2007). Influenza seasonality: underlying causes and modeling theories.. J Virol.

[21] Carstensen B (2007). Age-period-cohort models for the Lexis diagram.. Stat Med.

[22] CDC (1990). Guidelines for investigating clusters of health events.. MMWR Recomm Rep.

[23] Olson DR, Heffernan RT, Paladini M, Konty K, Weiss D (2007). Monitoring the impact of influenza by age: emergency department fever and respiratory complaint surveillance in New York City.. PLoS Med.

[24] Simental L, Martinez-Urtaza J (2008). Climate patterns governing the presence and permanence of salmonellae in coastal areas of Bahia de Todos Santos, Mexico.. Appl Environ Microbiol.

[25] Kovats RS, Edwards SJ, Hajat S, Armstrong BG, Ebi KL (2004). The effect of temperature on food poisoning: a time-series analysis of salmonellosis in ten European countries.. Epidemiol Infect.

[26] Chui KK, Webb P, Russell RM, Naumova EN (2009). Geographic variations and temporal trends of Salmonella-associated hospitalization in the U.S. elderly, 1991–2004: a time series analysis of the impact of HACCP regulation.. BMC Public Health.

[27] Altekruse SF, Street DA, Fein SB, Levy AS (1996). Consumer knowledge of foodborne microbial hazards and food-handling practices.. J Food Prot.

[28] Wenger JB, Naumova EN (2010). Seasonal synchronization of influenza in the United States older adult population.. PLoS One.

[29] Cohen SA, Klassen AC, Ahmed S, Agree EM, Louis TA (2010). Trends for influenza and pneumonia hospitalization in the older population: age, period, and cohort effects.. Epidemiol Infect.

[30] Petridou ET, Kyllekidis S, Jeffrey S, Chishti P, Dessypris N (2007). Unintentional injury mortality in the European Union: how many more lives could be saved?. Scand J Public Health.

[31] Rivera F, Lopez-Gomez JM, Perez-Garcia R (2002). Frequency of renal pathology in Spain 1994–1999.. Nephrol Dial Transplant.

[32] Styrke J, Stalnacke BM, Sojka P, Bjornstig U (2007). Traumatic brain injuries in a well-defined population: epidemiological aspects and severity.. J Neurotrauma.

[33] Taylor JO, Cornoni-Huntley J, Curb JD, Manton KG, Ostfeld AM (1991). Blood pressure and mortality risk in the elderly.. Am J Epidemiol.

[34] CDC (2004). Preliminary FoodNet data on the incidence of infection with pathogens transmitted commonly through food—selected sites, United States, 2003.. MMWR Morb Mortal Wkly Rep.

[35] Doshi M, DeMaria A, Naumova E (2007). Enteric Disease Surveillance: Seasonal Changes in Population Profiles.. Advances in Disease Surveillance.

[36] Amin OM (2002). Seasonal prevalence of intestinal parasites in the United States during 2000.. Am J Trop Med Hyg.

[37] Barwick RS, Levy DA, Craun GF, Beach MJ, Calderon RL (2000). Surveillance for waterborne-disease outbreaks—United States, 1997-1998.. MMWR CDC Surveill Summ.

[38] Bean NH, Goulding JS, Lao C, Angulo FJ (1996). Surveillance for foodborne-disease outbreaks—United States, 1988-1992.. MMWR CDC Surveill Summ.

[39] Naumova E, Christodouleas J, Hunter P, Syed Q (2005). Effect of precipitation on seasonal variability in cryptosporidiosis recorded by the North West England surveillance system in 1990–1999.. J Water Health.

[40] Patz JA, Engelberg D, Last J (2000). The effects of changing weather on public health.. Annu Rev Public Health.

[41] Naumova EN (2006). Mystery of seasonality: getting the rhythm of nature.. J Public Health Policy.

